# Liposome-based DNA carriers may induce cellular stress response and change gene expression pattern in transfected cells

**DOI:** 10.1186/1471-2199-12-27

**Published:** 2011-06-10

**Authors:** Anna Fiszer-Kierzkowska, Natalia Vydra, Aleksandra Wysocka-Wycisk, Zuzana Kronekova, Michał Jarząb, Katarzyna Marta Lisowska, Zdzisław Krawczyk

**Affiliations:** 1 Center for Translational Research and Molecular Biology of Cancer (previously Department of Tumor Biology), Maria Skłodowska-Curie Memorial Center and Institute of Oncology, Gliwice Branch, ul. Wybrzeże Armii Krajowej 15, 44-101 Gliwice, Poland; 2 Regional Blood Center, Tissue Bank Department, ul. Raciborska 15, 40-074 Katowice, Poland; 3 Polymer Institute, Slovak Academy of Sciences, Dubravska cesta 9, 845 41 Bratislava 45, Slovak Republic; 4 Department of Organic, Bioorganic Chemistry and Biotechnology, Silesian Technical University, ul. Krzywoustego 4, 44-100 Gliwice, Poland

## Abstract

**Background:**

During functional studies on the rat stress-inducible *Hspa1b *(*hsp70.1*) gene we noticed that some liposome-based DNA carriers, which are used for transfection, induce its promoter activity. This observation concerned commercial liposome formulations (LA), Lipofectin and Lipofectamine 2000. This work was aimed to understand better the mechanism of this phenomenon and its potential biological and practical consequences.

**Results:**

We found that a reporter gene driven by *Hspa1b *promoter is activated both in the case of transient transfections and in the stably transfected cells treated with LA. Using several deletion clones containing different fragments of *Hspa1b *promoter, we found that the regulatory elements responsible for most efficient LA-driven inducibility were located between nucleotides -269 and +85, relative to the transcription start site. Further studies showed that the induction mechanism was independent of the classical HSE-HSF interaction that is responsible for gene activation during heat stress. Using DNA microarrays we also detected significant activation of the endogenous *Hspa1b *gene in cells treated with Lipofectamine 2000. Several other stress genes were also induced, along with numerous genes involved in cellular metabolism, cell cycle control and pro-apoptotic pathways.

**Conclusions:**

Our observations suggest that i) some cationic liposomes may not be suitable for functional studies on *hsp *promoters, ii) lipofection may cause unintended changes in global gene expression in the transfected cells.

## Background

Transient transfection is widely used for functional studies on a gene of interest, eg. to study mechanisms regulating gene expression or functions, and cellular localization of its product. However, transfection is not neutral to such experimental models. In our previous studies we found that some transfection agents may induce by themselves the activity of a studied gene [1]. Moreover, high throughput methods of gene expression analysis revealed that transfection agents may induce not only a gene of interest but also a wide spectrum of other genes [2].

Our previous study was aimed to functionally characterize the heat inducible stress gene *Hspa1b *cloned by us from the rat genome (traditional name: *hsp70.1*; [3]). Endogenous *Hspa1b *is not transcribed at a physiological temperature. However, it is highly inducible by heat shock and many other harmful factors that cause accumulation of damaged proteins and/or fluidity changes of cellular membranes [3-6]. To analyze the *cis*-acting elements of *Hspa1b *gene promoter we used transient transfection by DEAE-Dextran method. This method gave reproducible results and a stable and expected pattern of *Hspa1b *promoter activity: undetectable or very low at physiological temperature and highly induced at heat shock temperature. However, with the advent of cationic liposomes, they were recognized as more efficient and more convenient than the previously used transfection agents [7,8]. Our attempts to use novel DNA carriers resulted in an unexpected finding. Indeed, Lipofectin had a higher transfection efficiency, but surprisingly it had also a side effect. It appeared that when using this DNA carrier the *Hspa1b *promoter contained in the transfected construct was induced and highly active, both under the heat shock conditions and at the physiological temperature [1]. Similar effects were seen when using Lipofectamine 2000. To our knowledge, this topic has not been studied since our first account. Our current work was aimed to better understand the mechanism of this phenomenon and its potential biological and practical consequences. Here we report comparisons of several transfection agents in respect to the described side effect, and our observations concerning the mechanism of transcriptional activation of *Hspa1b *by Lipofectin/Lipofectamine (LA). In addition, we show the microarray analysis of cellular processes and signalling pathways that are affected in the cells treated with Lipofectamine.

## Results

### The effect of cationic liposomes on *Hspa1b *gene transcription

**Figure 1:**
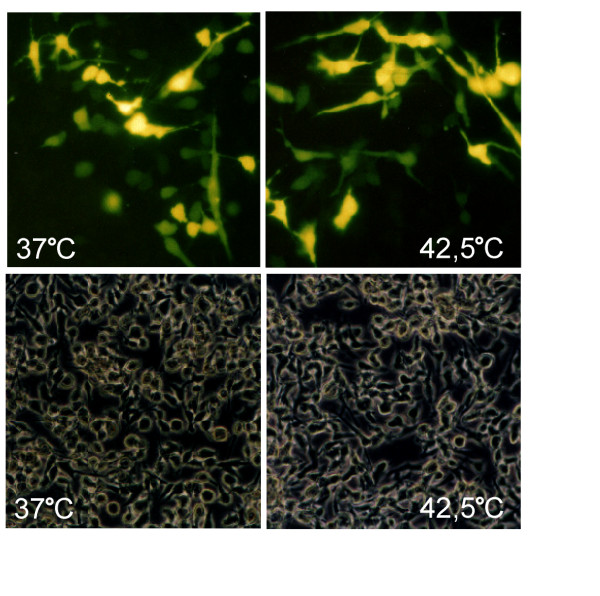
**The promoter of heat shock/stress gene *Hspa1b *is activated in physiological temperature when Lipofectin is used for transient transfection**. Mouse melanoma B16F10 cells were transfected with pR70/GFP vector containing the rat *Hspa1b *gene promoter linked with EGFP reporter gene. Left panel: cells grown at 37°C for 48 h after lipofection. Right panel: cells grown at 37°C for 24 h, then heat-shocked for 45 min at 42,5°C followed by recovery for 24 h at 37°C. Upper row - EGFP expression observed in UV light; lower row - the same field of vision observed in visible light. Pictures were recorded with an image analyzer equipped with Hamamatsu Color Chilled 3 CCD. Magnification: 250 x.

In a previous study we used Lipofectin for transient transfection of B16F10 mouse melanoma cells, with a construct containing a EGFP reporter gene controlled by the *Hspa1b *gene promoter. Surprisingly, we observed the reporter gene activity not only under heat shock conditions but also at the physiological temperature (Figure 1). Also, the B16F10 cells stably transfected with that construct showed EGFP activation when they were treated with Lipofectin [1]. This effect was observed repeatedly with different batches of the reagent.

**Figure 2:**
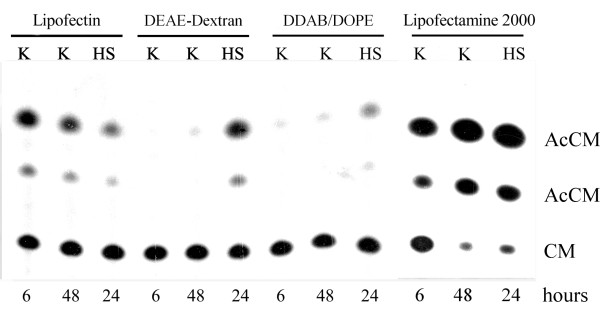
**Comparison of activation of *Hspa1b *promoter by different transfection agents (CAT assay)**. The B16F10 cells were transfected with p950/CAT6 construct containing *Hspa1b *promoter linked with CAT reporter gene using: Lipofectin (Gibco), DEAE-Dextran (Pharmacia), DDAB/DOPE (home made) and Lipofectamine 2000 (Invitrogen). K - cells grown at 37°C for 6 or 48 h after transfection. HS - heat-shocked cells, grown at 37°C for 24 h after transfection, then heat shocked at 42,5°C for 45 min followed by recovery for 24 h at 37°C. AcCM - acetylchloramphenicol, product of the enzymatic assay. CM - non-acetylated chloramphenicol, substrate of the assay.

For quantitative measurements we used the p950/CAT6 construct containing the bacterial chloramphenicol acetyltransferase (CAT) reporter gene under the control of *Hspa1b *promoter. We compared transfection efficiency and promoter activity dependence on various DNA carriers such as DEAE-Dextran, two home made liposome formulations (DDAB/DOPE mixture [9] and Arg-Chol/DOPE [10]) along with two commercially available liposome agents, Lipofectin and Lipofectamine 2000 (in the further text we use common abbreviation LA where the observations concern both agents; Lipofectamine 2000 is referred to as Lipofectamine). DDAB/DOPE and Arg-Chol/DOPE showed efficiency similar to that of DEAE-Dextran (usually between 5-10%, depending on the experimental setting, e.g. cell type and plasmid used for transfection). Lipofectin showed higher transfection efficiency (9-21%) while Lipofectamine was the most efficient (15-37%). When cells were transiently transfected with p950/CAT6 construct using DEAE-dextran, DDAB/DOPE or Arg-Chol/DOPE, they did not exhibit CAT activity at 37°C (physiological temperature) while at 42,5°C (heat shock) they did (Figure 2; data concerning Arg-Chol/DOPE is not shown; for more results obtained using DEAE-Dextran see also Figure 6 in [11]). This corresponds to the pattern of *Hspa1b *gene activity induced *in vivo *by heat shock. On the contrary, when LA were used for transfections, the *Hspa1b *promoter appeared to be equally active in control and in heat-shocked cells. In some cases CAT activity induced by these agents was even stronger at 37°C than under heat shock (Figure 2). This effect did not depend on transfection efficiency. It was observed repeatedly, although its intensity varied slightly between different batches of reagents.

We performed the same experiment using the rat hepatoma FTO cell line and observed the same effect of LA induced *Hspa1b *promoter activity at the physiological temperature (not shown).

**Figure 3:**
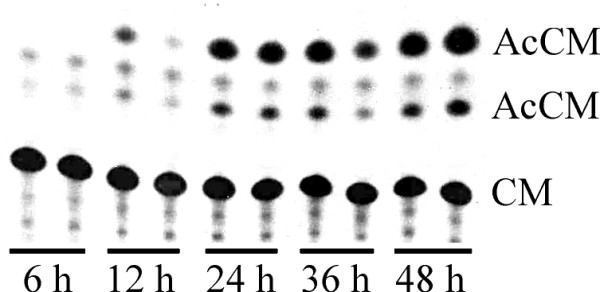
**Kinetics of *Hspa1b *promoter induction after transfection with Lipofectine (CAT assay)**. Chloramphenicol acetyltransferase (CAT) activity was observed at different time points after transfection with Lipofectine of p950/CAT6 construct into B16F10 cells. Each experimental point was performed in duplicate. AcCM - acetylchloramphenicol, product of the enzymatic assay. CM - non-acetylated chloramphenicol, substrate of the assay.

In the next step of the study we analyzed the kinetics of promoter activation by LA. B16F10 cells were transiently transfected with the p950/CAT6 construct and activity of the reporter gene was assayed at various time points up to 48 hours after transfection. In the cells grown at the physiological temperature, a high level of CAT activity was detected starting from 12 hours post transfection (Figure 3).

### Induction of transcription does not depend on HSE-HSF interaction

**Figure 4:**
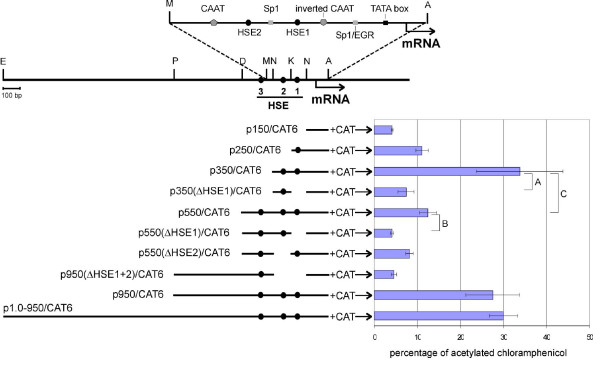
**Search for promoter fragment of the *Hspa1b *gene responsible for LA inducibility**. Left panel: Schematic representation of the plasmids used for transfections. Different fragments of the promoter and further upstream region of the rat *Hspa1b *gene were cloned in pBL-CAT6 vector. Upper part: Structure of analyzed DNA region. Restriction sites used for subcloning are shown (E - EcoR I, P - Pst I, D - Dra II, M - MaeIII, N - Nco I, K - Kpn I, A - Ava I). HSE - heat shock element. Region between -209 and +84 containing important regulatory elements is shown in details. Right panel: Data from transfection experiments. Plasmids were transiently transfected to the mouse B16F10 cells using Lipofectin. CAT activity was determined 24 hours after lipofection in cell extracts. CAT activity is shown as percentage of acetylated chloramphenicol. Each value is expressed as a mean (with the standard deviation) of four transfections. Brackets A, B and C were used to visualise the comparisons that are discussed in the text.

Ten plasmid constructs (Figure 4) containing the CAT gene under the control of different fragments of the *Hspa1b *gene promoter were used in searching for the mechanism responsible for LA-induced transcriptional activation. The CAT assay results (Figure 4) reflect the complexity of the transcriptional regulation in response to LA. The highest CAT activity was found with the plasmid p350/CAT6, which contains sequences localized between nucleotides -269 and +85, relative to the transcription start site of the *Hspa1b *gene. From among known regulatory sequences this DNA region contains TATA-box, two Sp1 binding sites, two CAAT-boxes (one inverted), Egr1 binding site and two proximal HSE sequences. The promoter fragment crucial for LA induction was located between nucleotides -269 and -64. Constructs lacking this DNA fragment always had their reporter gene activity reduced almost to the control level (see plasmid p150/CAT6 and plasmid p350ΔHSE1/CAT6, as compared to p350/CAT6 or plasmid p550ΔHSE1/CAT6, as compared to p550/CAT6). The DNA region localized between positions -269 and -478 confers unknown inhibitory abilities resulting in lower CAT activity (compare plasmid p550/CAT6 with p350/CAT6). Interestingly, this inhibition was reversed when the construct was enlarged by the addition of more upstream sequences (nt. from -478 to -869): CAT activity observed after transfection with the plasmids p950/CAT6 and p1.0-950/CAT6 was similar to that achieved with p350/CAT6.

**Figure 5:**
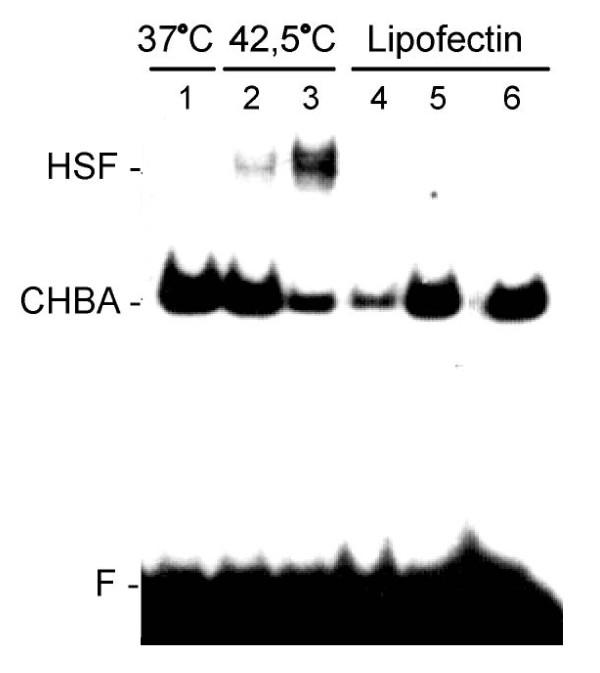
**Lack of the HSE-HSF interaction in LA treated B16F10 cells**. Nuclear extracts were prepared from B16F10 cells maintained at 37°C (lane 1), incubated at 42,5°C for 0,5h (2) and 1h (3) or treated with Lipofectin (Gibco) followed by recovery: 30 min (4), 1h (5), 2h (6). CHBA - constitutive HSE-binding activity corresponding to a Ku protein-containing complex; F - free HSE oligonucleotide.

It is generally accepted that promoter activation of heat shock genes by different stressful stimuli is mediated by the interaction of Heat Shock Transcription Factor 1 (HSF1) with a specific DNA sequence called HSE (Heat Shock Element) [4,5]. To verify whether this mechanism is employed during *Hspa1b *promoter induction by LA we performed an electrophoretic mobility shift assay (EMSA) using the oligonucleotide probe with canonical HSE sequence. In contrast to the heat-shocked cells, the nuclear extracts from the Lipofectin-treated cells did not exhibit any binding ability for the HSE sequence (Figure 5). This indicates that LA induction is independent from HSE-HSF interaction as well as from any other HSE-protein interaction.

### The influence of cationic liposomes on global gene expression

Inducible *hsp70 *gene expression is a hallmark of cellular stress response. In order to study how lipofection may affect cellular homeostasis we analyzed, using Affymetrix microarrays, the whole transcriptome of the mouse B16F10 cells treated with Lipofectamine. To find out which genes are affected we compared the cells treated with Lipofectamine, heat shocked cells and control, untreated cells. All three groups were compared by F-test (ANOVA), then two-way comparisons were carried out. In Lipofectamine-treated cells 419 probesets (representing 384 genes) showed significantly changed expression in comparison to the control, untreated cells. A similar number of 457 probesets (421 genes) were affected in heat shocked cells. Of these, 66 probesets were common for the heat-shocked and Lipofectamine-treated cells, all of them showing expression changes in the same direction. Lipofectamine induced several heat shock genes: the *Hspa1b *(*hsp70.1*) as well as *Hsph1 *(*hsp105*), *Hspa8 *(*hsc70*) and *Dnajb4*. In addition, Lipofectamine affected also *Hspa4 *(*hsp70RY*) which was the only *hsp *gene that showed a reduced level of transcription in comparison to the control cells. All these genes, except for the *hspa4 *(*hsp70RY*), were also affected by hyperthermia. An additional five *hsp *genes were upregulated by heat shock, but not by Lipofectamine. The comparison of *hsp *genes induced by Lipofectamine and by heat shock as well as the overall numbers of affected genes are given respectively in Tables 1 and 2.

**Table 1:** Comparison of a spectrum of stress genes (*hsp *family) affected by Lipofectamine 2000 versus heat shock in B16F10 cells

Gene	LA	HS
*Hspa1b*	+	+
*Hsph1 (hsp105)*	+	+
*Hspa8 (hsc70)*	+	+
*Dnajb4*	+	+
*Hspa4 (hsp70RY)*	-	
*Hspa1a (hsp70.3)*		+
*Hspb1 (hsp27)*		+
*Hspa4l (94kDa osmotic stress protein)*		+
*Hspe1 (hsp10)*		+
*Hsph1 (hsp110)*		+

LA - Lipofectamine 2000, HS - heat shock, "+" - increased expression compared to control, untreated cells, "-" - decreased expression, blank - no change.

**Table 2:** Comparison of a number of probsets affected by Lipofectamine 2000 and heat shock in B16F10 cells

	Probesets affected	Up	Down
LA	419	161	257
HS	457	89	366
Both	66		

LA - Lipofectamine 2000, HS - heat shock, "Up" - number of upregulated probesets, "Down" - number of downregulated probsets (Affymetrix), "Both" - number of probsets affected by both factors.

As expected, heat shock caused widespread suppression of genes transcription and specific induction of cytoprotective genes and pro-survival signalling pathways. Lipofectamine, despite inducing some stress genes, evoked pro-apototic rather than pro-survival signalling. Numerous affected genes were involved in general metabolism, cell cycle control and progression, as well as apoptosis. Most significantly downregulated gene ontology classes were those engaged in cell proliferation, viability and metabolism, e.g. classes named "DNA-dependent DNA replication", "double-strand break repair", "chromosome segregation", "ribosome biogenesis". Significantly upregulated were classes such as "regulation of caspase activity", "regulation of peptidase activity" or "stress-activated protein kinase signalling pathway".

### Promoter analysis of liposome- and heat shock-induced genes

For promoter analysis we used the Molecular Signatures Database, MSigDB and its Predicted Promoter Motifs ontology which consists of a sets of genes that share a common transcription factor binding site. Analysis showed that among the genes upregulated by Lipofectamine, overrepresented were those that contained in their promoters a binding site for Serum Response Factor (SRF), eg. EGR1, EGR2, FOS, JUNB, GADD45G, DNAJB4, TLE3 and DUSP6. Other promoter sites indicated by this analysis included: a binding site for Aryl Hydrocarbon Receptor (AHR), a sequence recognized by microRNA miR-191 and one motif that does not match any known transcription factor but is present in the promoters of 78 genes included in MSigDB. In contrast, many more types of regulatory sequences were ascribed to the genes induced by heat shock, including HSF1, HSF2, one uncharacterized motif resembling a HSF-binding element, binding sites for transcription factors TCF8, TCF3 and E2F1, as well as for hormone receptors PR and AR and motifs recognized by four microRNAs (526C, 518F, 526A, 153).

## Discussion

### *Hsp70 *induction by LA - unique observation or universal phenomenon?

Although *hsp70 *gene promoter induction by Lipofectin or Lipofectamine was not reported until our first account [1], the Pub Med search revealed several examples of similar observations concerning *hsp70 *genes from different species (mouse and *Drosophila*), transfected into different cell types (from chickens and insects). These observations were, however, overlooked or misinterpreted by the authors. Brazolot et al. aimed to establish conditions for efficient lipofection (Lipofectin, Bethesda Research Laboratories) for two types of chicken cells (blastodermal and primary fibroblasts) [12]. For this purpose they chose the construct containing lacZ reporter gene under the control of murine *hsp68 *promoter. They noticed that expression of this construct did not require induction by heat shock, but they ascribed this effect to the heterology of the experimental model consisting of chicken cells and murine promoter. In our opinion this was the effect of lipofectin-induced *hsp *promoter activation. In another study, Lan and Riddiford tried to optimize conditions for transient transfection of embryonic cell line GV1 from a moth *Manduca sexta *by comparing polybrene, CellFectin and Lipofectin [13]. When they used Lipofectin (GIBCO Laboratories) for transfection of the constructs containing *Drosophila hsp70 *promoter fused with a LacZ or CAT gene, they observed constitutive activity of the reporter genes at the physiological temperature. They also noticed higher reporter gene expression in heat shock conditions in the cells transfected by Lipofectin than by other transfection agents. Both effects were considered a result of higher transfection efficiency achieved with Lipofectin. We think that despite possible differences in transfection efficiency, these observations reflect the induction of *hsp70 *promoter by Lipofectin. Stiles and co-workers transfected *hsp70*-CAT construct to the boll weevil cells using Lipofectin (BRL) and they noted that, even without heat shock, the *Drosophila Hsp70 *promoter provided some CAT expression up to two days after transfection [14]. Comparing with the heat shock, the level of CAT activity in control cells was rather minimal but it was comparable to the level achieved by CuSO_4 _induction.

Our data was derived from the studies done on two cell lines (mouse melanoma B16F10 and rat hepatoma FTO) and including *Hspa1b *gene derived from two species (the rat *Hspa1b *gene was used in promoter studies, activation of the mouse *Hspa1b *gene was detected by microarrays in an experiment done on the mouse B16F10 cells). In addition we observed induction of three other *hsp *genes by Lipofectamine in the microarray study. In our opinion, the results of our studies, together with the above-mentioned observations made by others suggest that the phenomenon of *hsp70 *promoter induction by Lipofectin may be universal, affecting *hsp70 *genes from different species and occurring in wide range of species and cell types.

### Not every liposome formulation induces *hsp *transcription

We have shown that Lipofectin and Lipofectamine have the same activity toward the rat *Hspa1b *promoter. The *Hspa1b *promoter induction in physiological temperature was observed consistently with both reagents, although the intensity of induction was slightly varying from batch to batch. It seems however that not every cationic liposome formulation induces *hsp70 *promoters. For example, we did not observe *Hspa1b *promoter induction when using laboratory-formulated liposomes DDAB/DOPE and Arg-Chol/DOPE [10]. Also, Boulo et al. who used DOTAP reagent (Boehringer) did not observe induction of the Drosophila *hsp70 *gene promoter that had been cloned into the transfected construct. The figure showing reporter gene activity at different temperatures, from 18°C (physiological) to 42°C (severe heat shock for these cells), clearly demonstrated that promoter induction was dependent only on temperature [15]. Comparison of the composition of different liposomal formulations and their inducing activity suggests that a neutral lipid DOPE is not engaged. DOPE was found both in those transfection agents that induce *hsp70 *promoter and those that do not. Therefore, the inducing factor must be a cationic liposome component. However, our results and the observations made by others suggest that not every type of cationic liposome formulation induces *hsp *transcription. In summary, DOTMA (contained in Lipofectin) and DOSPA (contained in Lipofectamine) have inducing ability while DDAB, Arg-Chol and DOTAP are devoid of *hsp70 *inducing activity.

### Promoter analysis in relation to LA induction

The inducible *hsp70 *genes code for evolutionarily conserved proteins that confer cytoprotection against a wide range of stressful factors. Their expression is induced by a number of various substances and conditions that lead to aggregation of misfolded proteins and/or to fluidity changes of cellular membranes. Transcription of stress inducible *hsp70 *genes is mediated by the HSF1 factor that interacts with HSE sequence. Apart from this interaction, which has been best-studied and considered basic, the expression of *hsp70 *genes may be modulated by other members of HSF family (HSF2, HSF3, HSF4); rev in: [4] as well as by unrelated transcription factors e.g. Sp1, MYB, TP53, STAT, MYC [5].

In this study we identified the minimal DNA fragment required for efficient liposome-induced response of *Hspa1b *gene. This DNA region (nucleotides from -269 to +84, according to the transcription start point) contains two CAAT-boxes (one inverted), one Sp1/EGR1 binding site and two HSE sequences. According to the results of the EMSA experiment, HSE sequences are not engaged in LA activation. This result was consistent with the results of the microarray experiment and data analysis according to the MSigDB, which did not indicate HSF1 motif in other LA-induced genes. Interestingly, this analysis indicated very few regulatory motifs potentially engaged in LA stimulation, none of them being present in the analyzed fragment of *Hspa1b *gene. However, when the two-step model of liposome-induced activation is considered, it is possible that Early Growth Response 1 (EGR1) protein that is induced by LA could be responsible for *Hspa1b *activation. Initially, we also speculated that LA induction of *Hspa1b *promoter could be triggered by an alteration of cellular membrane structure. It was shown recently that reorganization of cholesterol-rich plasma membrane microdomains, provoked either by high temperature or benzyl alcohol treatment, induces stress response mediated by the HSE-HSF1 interaction [16,17]. However, we experimentally excluded HSE-HSF1 mechanism, thus it seems that membrane perturbations do not play a key role during cellular response to LA.

### Transfection agents may cause widespread transcriptional response

We also got an insight into molecular changes caused by Lipofectamine. Using DNA microarrays we found that in mouse B16F10 cells, except several *hsp *genes, numerous other genes were affected. The majority of them are engaged in vital cellular functions, mostly maintaining cellular metabolism, cell cycle control and progression, and apoptosis. In our opinion, the observed alterations in gene expression profile are consistent with phenotypic changes caused by LA *in vitro*. These agents are cytotoxic and high concentrations or prolonged incubation lead to cell death. The results of our study show that also the apparently non-lethal concentrations trigger cellular alert signals and widespread transcriptional response.

The question of unintended changes in gene expression induced by different DNA carriers was also addressed by Jacobsen et al. [2]. However, their experiment differed from ours, as they transfected a gene of interest while we did only a mock transfection. Thus, we observed the changes induced exclusively by a transfection agent while they observed a joint effect of a transfected DNA and a DNA carrier. Probably for this reason they did not analyze the biological effects of each transfection agent. Nevertheless, they noticed variable numbers of affected genes, depending on the transfection reagent used. Eight hours post transfection these were: 4 transcripts in the case of FuGene HD (Roche), 25 transcripts for Effectene (Qiagen), 39 transcripts for Lipofectamine LTX used with PLUS Reagent (Invitrogen) and 68 transcripts for Lipofectamine 2000 (Invitrogen) (in comparison to untransfected controls). After 48 hours there were: 157, 1643, 556 and 1908 affected genes respectively for the aforementioned transfection agents. The authors emphasize that, except for biologically relevant changes caused by the gene of interest, they observed both universal cellular response to the foreign DNA and a differential effect of each transfection agent. Thus, by employing microarray technique, which has a high throughput and is a highly sensitive method, it is possible to measure widespread changes in global gene expression, caused by transfection agents. This may be of particular importance when searching for gene function through transient overexpression or silencing.

## Conclusions

The results of our study indicate that LA cause activation of the rat stress inducible *Hspa1b *gene. In addition, we observed that Lipofectamine induced expression changes of the mouse *Hspa1b *gene and other stress genes as well as many unrelated ones in the B16F10 cells. We postulate that these transfection agents may not be suitable for functional studies of *hsp70 *promoters.

We are also convinced that the phenomenon of widespread gene expression changes caused by transient transfection should be further studied as it may possibly affect the results of *in vitro *gene function studies done with microarrays.

## Methods

### Cell culture and treatment

The mouse melanoma B16F10 cells were grown in RPMI (Sigma) medium supplemented with 10% fetal bovine serum (FBS). Heat shock was performed at 42,5°C. For the EMSA experiment duration of heat shock was 30 min and 1 h, without recovery at 37°C. For microarray analysis duration of heat shock was 1 h at 42,5°C with 30 min recovery at 37°C. LA treatment was done as described in "Transfections" but without adding plasmid DNA. For the EMSA experiment cells were treated with Lipofectin (Gibco) or Lipofectamine 2000 (Invitrogen) in Opti-MEM (Gibco BRL) medium for 1 h and 2 h and processed immediately. For microarray analysis of LA effect, cells were treated with Lipofectamine 2000 (Invitrogen) for 3 h in Opti-MEM (Gibco BRL) medium and then the liposomes containing medium was replaced with RPMI supplemented with 10% FBS and cells were allowed to recover for 30 min at 37°C.

### Transfections

The transient transfections have been performed using the following compounds: DEAE-Dextran (Pharmacia), Lipofectin (1:1 DOTMA and DOPE, Gibco BRL), Lipofectamine 2000 (3:1 DOSPA and DOPE, Invitrogen) and two liposome formulations prepared in our Institute DDAB/DOPE [9] and Arg-Chol/DOPE [10].

For transient transfection using DEAE-Dextran, cells were seeded at 3 × 10^5 ^cells on a 90 mm plate. The next day, cells were treated with the mixture containing 10 μg plasmid DNA and 1 mg/ml DEAE-Dextran in TBSP buffer (25 mM Tris-HCl, pH 7.4, 137 mM NaCl, 5 mM KCl, 0.7 mM CaCl_2_, 0.5 mM MgCl_2 _and 1 mM Na_2_HPO_4_) at room temperature for 30 min. Then cells were washed with a TBSP buffer and treated with RPMI with 10% DMSO for 2 min. Cells were washed with DMSO-free TBSP buffer, then medium supplemented with FBS was added and cells were incubated at 37°C in a CO_2 _incubator for 6-48 h.

Transient transfections using liposome formulation DDAB/DOPE, Arg-Chol/DOPE and Lipofectin were done according to standard procedure. Cells were seeded at 2 × 10^5 ^cells on 60 mm plate a day before. For each transfection, 10 μg plasmid DNA was diluted in 100 μl Opti-MEM medium (Gibco BRL) and added to liposome dilution. Liposome dilution was prepared by adding 20 μl of liposomes (1 mg/ml) into 100 μl Opti-MEM and followed by incubation at room temperature (RT) for 45 min. DNA-liposome mixture was incubated for 15 min at RT. While complexes were forming, the cells were washed twice with serum-free Opti-MEM medium and then overlaid with that medium. DNA-liposome complexes were dropped onto the plate. Cells were incubated at 37°C in a CO_2 _incubator for 3 h. Then the medium was changed for RPMI supplemented with FBS and cells were incubated at 37°C in a CO_2 _incubator. Cells were either grown for 6 or 48 h, or after 24 h at 37°C heat shocked for 45 min at 42,5°C followed by recovery for 24 h. For kinetics study, incubation times were 6 - 48 h.

Transient transfection using Lipofectamine 2000 was done according to the supplier's instruction. Cells were seeded at 1,2 × 10^5 ^cells on a 30 mm plate a day before. For each transfection, 4 μg of plasmid DNA was diluted in 250 μl of Opti-MEM and added to liposome dilution. Liposome dilution was prepared by adding 10 μl of Lipofectamine 2000 into 250 μl Opti-MEM and following incubation at RT for 5 min. DNA-liposome mixture was incubated for 30 min to allow formation of DNA-liposome complexes. The next steps were done as described above for transient transfection using Lipofectin and other liposome formulation.

### Plasmid constructs

Constructs used for functional analysis of the rat *Hspa1b *promoter contained 85 bp of the 5'UTR and different fragments of the promoter region, cloned in pBL-CAT6 vector bearing the bacterial chloramphenicol acetyltransferase (CAT) reporter gene (Figure 4) [11]. pR70/GFP plasmid contained PstI-AvaI restriction fragment (from nt -870 to nt +85) of the rat *Hspa1b *promoter cloned into the SalI and BglII sites of pEGFP-1 Promoter Reporter Vector (Clontech) [1].

### Electrophoretic mobility shift assay

Nuclear extracts were prepared as described by Suzuki et al. [18]. Nuclei isolated from cells were lysed by Nonidet-P40 and proteins were extracted with buffer consisting of 0.35 M NaCl, 5 mM EDTA, 1 mM DTT, 10 mM Hepes, pH 7.9 and 0.2 mM PMSF by incubation on ice for 20 min. DNA-protein binding activity was analyzed by incubating nuclear extracts with [α-32P]-labeled double stranded HSE oligonucleotide (HSE consensus sequence):

5'-gcTTCtaGAAgcTTCctGAAgcTTCtaGAA- 3'

3'-cgAAGatCTTcgAAGgaCTTcgAAGatCTT- 5'.

Binding buffer contained 20 mM Tris-HCl, pH 7.6, 5 mM MgCl_2_, 1 mM DTT, 5% glycerol, 1 mM EDTA, 0.2 M NaCl, 2 μg poly(dI-dC). The HSE-protein complexes were separated on a native 6% polyacrylamide gel. Gels were dried and exposed to an X-ray film (Kodak).

### CAT Assay

Cell culture extracts were prepared by the Tris buffer freeze-thaw protocol. Protein concentration was determined by Bradford method. The CAT activity was detected as described in [18]. Briefly, 25 μg of protein from cell extracts were incubated with ^14^C-labeled chloramphenicol (ICN) and acetyl-coenzyme A (Sigma) for 4 h at 37°C. The reaction products were separated by thin layer chromatography, then plates were exposed to X-ray film (Kodak). For quantitative analysis, CAT activity was monitored by scintillation counting; counts were converted to the percentage of acetylated chloramphenicol as described in [19]. Data for each experimental point was gathered in quadruplicate.

### RNA isolation

Total RNA was isolated using RNeasy Mini Kit (Qiagen) with on-column DNA digestion using DNAse I (Qiagen) according to the manufacturer's recommendations. RNA quantity was estimated with ND-1000 spectrophotometer (NanoDrop Technologies). RNA quality was assessed using Agilent platform: RNA 6000 Nano LabChip Kit, RNA Integrity Number software and the Agilent 2100 Bioanalyzer (Agilent Technologies).

### Oligonucleotide microarrays

We used the Mouse Expression Arrays 430A (Affymetrix). The hybridization target was prepared according to the recommendations from microarrays' manufacturer. Total RNA (8 μg) was used for synthesis of double stranded cDNA. Half of the cDNA volume was used for synthesis of biotinylated cRNA with the BioArray High Yield RNA Transcript Labeling Kit (Enzo Diagnostics). Both cDNA and cRNA were purified with Gene Chip Sample Cleanup Module (Affymetrix). cRNA (16 μg) was fragmented and hybridized to the microarray for 16 h at 45°C. After washing and staining, the microarrays were scanned with GeneChip Scanner 3000 (Affymetrix). Data was acquired using GCOS 1.2 software (Affymetrix). The preprocessing was performed by Robust Multiarray Analysis (RMA, Bioconductor). Microarray data are available via NCBI GEO: http://www.ncbi.nlm.nih.gov/geo/query/acc.cgi?acc=GSE29743.

### Data analysis

Supervised gene selection was carried out by BRB Array Tools (developed by R. Simon and BRB Tools Development Team). Genes differentiating three analyzed groups were selected using a random-variance F-test. A stringent significance threshold was used to limit the number of false positive findings: differences in the expression of genes were considered statistically significant if their p value was less than 0.001. We also performed a global test of whether the expression profiles differed between the classes by permuting the labels of which arrays corresponded to which classes. For each permutation, the p values were re-computed and the number of genes significant at the 0.001 level was noted. The proportion of the permutations that gave at least as many significant genes as with the actual data was the significance level of the global test. False Discovery Rate (FDR) was estimated by Benjamini-Hochberg algorithm. Gene group analysis was carried out to obtain p values reflecting the differential expression of gene groups among classes. Four different tests: the Fisher (LS) statistic, Kolmogorov-Smirnov (KS) test, GSA test and Goeman test were used, as implemented in BRB Array Tools. We considered a GO category significantly differentially regulated if either significance level was less than 0.005, all categories with between 5 and 100 genes represented on the array were considered; some of the groups were overlapping. Promoter analysis was carried out using the Molecular Signatures Database, MSigDB. The MSigDB Predicted Promoter Motifs ontology contains sets of genes that share a transcription factor binding site in their promoters. The binding sites are predicted based on definitions from the TransFac database.

## Authors' contributions

AFK constructed most of the recombinant plasmids, carried out promoter analysis by CAT assay, NV constructed some plasmids, analyzed the kinetics of LA induced *Hspa1b *promoter activity and carried out the treatments of the cells for microarray experiment, AW did the EMSA experiment and transfections with pR70/GFP plasmid, ZuK did microarray experiments, MJ analyzed the microarray data, KL constructed some plasmids, drafted the manuscript, participated in the design of the study, ZK conceived the study, participated in its design and coordination and helped to draft the manuscript. All authors read and approved the final manuscript.
